# Brain region-specificity of palmitic acid-induced abnormalities associated with Alzheimer's disease

**DOI:** 10.1186/1756-0500-1-20

**Published:** 2008-06-04

**Authors:** Sachin Patil, Deebika Balu, Joseph Melrose, Christina Chan

**Affiliations:** 1 Department of Chemical Engineering and Material Science, Michigan State University, East Lansing, Michigan, USA; 2 Department of Biochemistry and Molecular Biology, Michigan State University, East Lansing, Michigan, USA

## Abstract

**Background:**

Alzheimer's disease (AD) is a progressive, neurodegenerative disease mostly affecting the basal forebrain, cortex and hippocampus whereas the cerebellum is relatively spared. The reason behind this region-specific brain damage in AD is not well understood. Here, we report our data suggesting "differential free fatty acid metabolism in the different brain areas" as a potentially important factor in causing the region-specific damage observed in AD brain.

**Findings:**

The astroglia from two different rat brain regions, cortex (region affected in AD) and cerebellum (unaffected region), were treated with 0.2 mM of palmitic acid. The conditioned media were then transferred to the cortical neurons to study the possible effects on the two main, AD-associated protein abnormalities, viz. BACE1 upregulation and hyperphosphorylation of tau. The conditioned media from palmitic-acid treated cortical astroglia, but not the cerebellar astroglia, significantly elevated levels of phosphorylated tau and BACE1 in cortical neurons as compared to controls (47 ± 7% and 45 ± 4%, respectively).

**Conclusion:**

The present data provide an experimental explanation for the region-specific damage observed in AD brain; higher fatty acid-metabolizing capacity of cortical astroglia as compared to cerebellar astroglia, may play a causal role in increasing vulnerability of cortex in AD, while sparing cerebellum.

## Findings

Alzheimer's disease (AD) is a progressive neurodegenerative disease, which affects basal forebrain, cortex and hippocampus, while cerebellum is relatively spared [1]. AD brain is characterized by extracellular deposits of amyloid beta (Aβ) protein and intracellular accumulation of neurofibrillary tangles [1]. Aβ protein is derived from the proteolysis of β-amyloid precursor protein (APP) by β-secretase (BACE1) and γ-secretase, BACE1 step being the rate-limiting step [2]. The aggregation of Aβ leads to the formation of senile plaques, a characteristic feature of AD [1]. Aβ aggregates exert toxicity to nerve cells and thus play a central role in AD-associated neurodegeneration [1]. Neurofibrillary tangles, the other major characteristic of AD pathology, are composed of paired helical filaments of microtubule-associated tau protein, which is hyperphosphorylated in AD [3]. The hyperphosphorylation of tau leads to the disruption of the cytoskeleton of neurons and their degeneration, thus playing an important role in AD pathology [1].

Epidemiological studies implicate that high fat diet confers a significant risk for development of AD and the degree of saturation of fatty acids is critical in determining the risk for AD [4]. These studies are further supported by various animal studies where mice fed high-fat-high cholesterol diet developed AD-like pathophysiological changes in their brain [5,6]. Furthermore, various vascular diseases such as diabetes mellitus, hypertension and obesity, which are characterized by increased levels of saturated free fatty acids (FFAs), pose a significant risk for developing AD [7-9]. Also, traumatic brain injury is an independent risk factor for AD [10], and is characterized by elevated levels of saturated fatty acids in the brain [11], with palmitic acid increasing from ~60 to 180 μM and stearic acid from ~50 to 350 μM [12]. Finally, apolipoprotein E4 (ApoE4) is an important genetic risk factor for AD and its risk may be further increased by diets rich in saturated fats [13].

Despite these data suggesting causal involvement of saturated fatty acids in AD pathogenesis, their potential role in causing the region-specific damage associated with AD pathology is not well studied. In the present study, we report our data suggesting "differential free fatty acid (FFA) metabolism in the different brain areas" as a potential causal factor behind the region-specific damage observed in AD.

## Methods

### Isolation and culture of neurons and astroglia from rat cortex

Primary cortical neurons were isolated from one-day-old Sprague-Dawley rat pups and cultured according to the published methods as described by Chandler et al. [14]. The cells were plated on poly-l-lysine-coated, six-well plates at the concentration of 2 × 10^6 ^cells per well in fresh cortical medium [Dulbecco's Modified Eagle's Medium (DMEM, from Invitrogen, CA, USA) supplemented with 10% horse serum (Sigma, MO, USA), 25 mM glucose, 10 mM HEPES (Sigma), 2 mM glutamine (BioSource International, CA, USA), 100 IU/ml penicillin, and 0.1 mg/ml streptomycine]. To obtain pure neuronal cell cultures, the medium was replaced with the cortical medium supplemented with 5 μM cytosine-β-arabinofuranoside (Arac, from Calbiochem, CA) after 3 days of incubation (37°C, 5% CO2). After 2 days, the neuronal culture was switched back to cortical medium without Arac. The experiments were performed on 6- to 7-day-old neuronal cells. To obtain primary cultures of cortical astroglia, the cortical cells from one-day-old Sprague-Dawley rat pups were cultured in DMEM/Ham's F12 medium (1:1), 10% fetal bovine serum (Biomeda, CA, USA), 100 IU/ml penicillin, and 0.1 mg/ml streptomycine [15]. The cells were plated on poly-d-lysine coated, 6-well plates at the concentration of 2 × 106 cells per well. Cells were grown for 8–10 days (37°C, 5% CO2) and culture medium was changed every 2 days. Twenty-four hours prior to treatment with palmitic acid, the medium was changed to cortical neuronal cell culture medium.

### Isolation and culture of astroglia from rat cerebellum

The enzyme digestion and trituration techniques were performed on the cerebellum tissue from 7-day-old rat pups, as described previously [16]. Briefly, dissected cerebellar tissue was placed in a cerebellar buffer solution containing 136.89 mM NaCl, 5.36 mM KCl, 0.34 mM Na2HPO4, 0.44 mM KH2PO4, 5.55 mM dextrose, 20.02 mM Hepes, and 4.17 mM NaHCO3, PH 7.4. Cerebellar tissue was minced, transferred to 0.025% trypsin solution in cerebellar buffer, and incubated in water bath for 15 minutes at 37°C. 0.04% DNase I solution in cerebellar medium (DMEM supplemented with 10% horse serum, 25 mM KCl, 5 mg/ml insulin, 50 μM GABA, 100 IU/ml penicillin, and 0.1 mg/ml streptomycine) was then added to inactivate trypsin. After the supernatant was collected from the trituration steps, the cells were separated from the debris into 4% BSA solution in cerebellar buffer supplemented with 0.03% MgSO4. Finally, the cells were plated onto poly-l-lysine coated 6-well culture dishes at a density of 2.0 × 10^6 ^cells/ml in 3 ml of fresh cerebellar medium. After 2 days, the media was switched to DMEM/Ham's F12 medium (1:1) to obtain pure cultures of cerebellar astroglia. The astroglia cell cultures with approximately 95% purity were obtained after 8–10 days culture (37°C, 5% CO2). Twenty-four hours prior to treatment with palmitic acid, the medium was changed to cortical neuronal cell culture medium.

### Western blot analyses

For Western blotting, cells were washed three times with ice-cold TBS (25 mM Tris, pH 8.0, 140 mM NaCl, and 5 mM KCl) and lysed for 20 min by scraping into ice-cold lysis buffer. To extract membrane protein BACE1, lysis buffer containing 1% (v/v) Nonidet P-40, 0.1% (w/v) SDS, 0.5% (w/v) deoxycholate, 50 mM Tris, pH 7.2, 150 mM NaCl, 1 mM Na3VO4 and 1 mM PMSF [all chemicals from Sigma] was used [17]. For phosphorylated tau, lysis buffer containing 1% (v/v) Triton, 0.1% (w/v) SDS, 0.5% (w/v) deoxycholate, 20 mM Tris, pH 7.4, 150 mM NaCl, 100 mM NaF, 1 mM Na3VO4, 1 mM EDTA, 1 mM EGTA, and 1 mM PMSF [all chemicals from Sigma] was used [18]. The total cell lysate was obtained by centrifugation at 12,000 rpm for 15 min at 4°C. The total protein concentration was measured by BCA protein assay kit from Pierce (Rockford, IL, USA). Equal amounts of total protein from each condition were run at 200 V on 10% SDS-PAGE gels (BioRad, CA, USA). The separated proteins were transferred to nitrocellulose membranes for 1 h at 100 V and incubated at 4°C overnight with the appropriate primary antibodies [1:1000 BACE1 (Chemicon, CA, USA), 1:200 AT8 (Pierce Biotechnology, IL, USA), 1:2000 actin (Sigma)]. Blots were washed three times in PBS-Tween (PBS-T) and incubated with appropriate HRP-linked secondary antibodies (Pierce Biotechnology, IL, USA) diluted in PBS-T for 1 h. After an additional three washes in PBS-T, blots were developed with the Pierce SuperSignal West Femto Maximum Sensitivity Substrate (Pierce Biotechnology) and imaged with the BioRad ChemiDoc.

### Data Analyses

Data are shown as means ± S.D. for three independent experiments. Student's t-test was used to evaluate statistical significances between different treatment groups. Statistical significance was set at p < 0.05.

## Results and Discussion

**Figure 1:**
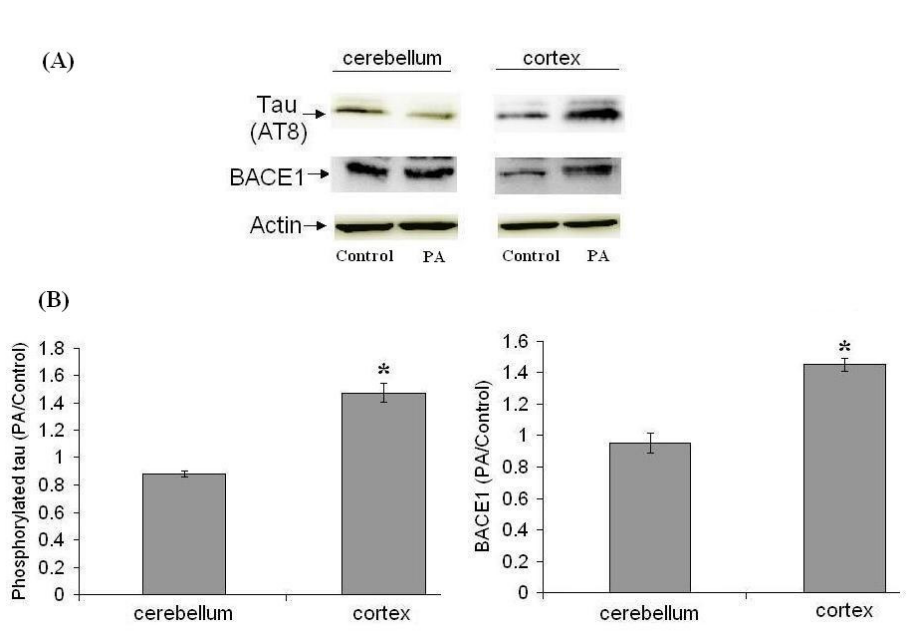
**Cortical, but not cerebellar, astroglia mediate FFA-induced tau hyperphosphorylation and BACE1 upregulation in cortical neurons**. Both cortical and cerebellar astroglia were treated for 24 hours with 0.2 mM of palmitic acid (PA) or 4% BSA (control), followed by transfer of the astroglia-conditioned media to cortical neurons (24 hours treatment). (A) Western blot analysis of hyperphosphorylated tau (AT8 antibody) and BACE1. β-actin is shown as a marker for protein loading. (B) Histograms corresponding to AT8 and BACE1 blots represent quantitative determinations of intensities of the relevant bands, presented as the ratio of PA-treated to control. Data represent mean ± S.D. of three independent experiments. Student's t-test was used for analyzing differences between two treatment groups (* p < 0.05 compared with control).

Previously, we showed that saturated fatty acids, palmitic acid (PA) and stearic acid (SA), at pathological concentrations (0.2 mM) have no direct effect on primary rat cortical neurons [19-21]. This may be attributed to the low capacity of primary neurons to take up and metabolize fatty acids [22]. The astroglia, in contrast, possess a relatively higher capacity to utilize fatty acids, leading to a significant upregulation in the *de novo *synthesis of ceramide when treated with saturated FFAs [21,22]. PA-induced ceramide synthesis in cortical astroglia plays a central role in causing tau hyperphosphorylation and BACE1 upregulation in cortical neurons, possibly through secretion of cytokines or other signaling molecules (e.g. nitric oxide) from astroglia [21]. Tau hyperphoshorylation and increased amyloidogenesis due to BACE1 upregulation leads to the formation of the two most important pathophysiological hallmarks of AD, neurofibrillary tangles and Aβ plaques, respectively. Based on these data, which place "astroglial FFA metabolism" at the centre of the pathogenic cascade leading to AD, we hypothesized that differential FFA metabolism by astroglia from different brain areas may be a deciding factor in the susceptibility of the different brain regions; the higher the FFA metabolic activity the greater the damage and vice versa. The hypothesis was based on the fact that the activity of fatty acyl-CoA synthetase (FACS) is 10× lower in cerebellum (unaffected region in AD) as compared to the other affected brain regions [23]; FACS is the first enzyme involved in FFA metabolism, which converts fatty acid to fatty acyl-CoA, which is then utilized by the cells in anabolic (e.g. ceramide synthesis) or catabolic (e.g. β-oxidation) pathways [24]. Thus, under pathologically elevated levels of saturated FFAs, cerebellum may be less likely to be affected by abnormal FFA metabolism due to its lower activity level of FACS as compared to the other affected brain regions (e.g. cortex). To test this hypothesis, we treated cortical and cerebellar astroglia for 24 hours with equal concentration of saturated fatty acid, 0.2 mM PA, or 4% bovine serum albumin (BSA, control) and then transferred the astroglia-conditioned media to cortical neurons. After 24 hours of treatment with astroglia-conditioned media, we checked the levels of phosphorylated tau and BACE1 in cortical neurons. The conditioned media from PA-treated cerebellar astroglia, did not affect levels of both phosphorylated tau and BACE1 in cortical neurons as compared to controls (Figure 1). The conditioned media from PA-treated cortical astroglia, on the other hand, induced significant tau hyperphosphorylation and BACE1 upregulation in cortical neurons as compared to the respective controls (47 and 45%, respectively). These data support our hypothesis, further emphasizing potentially causal role of astroglial FFA metabolism in AD pathogenesis and region-specific brain damage associated with it.

The major limitation associated with the present study may be the use of cortical and cerebellar astroglia isolated from different animals and by different techniques. The well-established protocols used in our studies for the isolation of rat cortical [14] and cerebellar [16] cells, required use of rat pups of different ages. For the isolation of rat cortical cells, embryonic [2] or very young, 1–2 day old [14] rat pups are preferred and thus 1-day old rat pups were used in our studies. The use of older rat pups for the isolation of rat cortical cells significantly decreases the viability and quality of the cells. On the other hand, 6–7 day old rat pups have been widely used for the isolation of cells from rat cerebellum [16,25]. The use of older rats for the isolation of cerebellar cells may be attributed to the fact that the cerebellum develops postnatally as compared with cortex [15]. Despite these differences, both cortical and cerebellar astroglia were allowed to proliferate and mature under similar conditions (cultured in the same medium for the same amount of time). Moreover, these confluent cultures of astroglia from both the brain areas were cultured in the same growth medium (cortical neuronal medium) 24 hours prior to treatment. Also, during the treatment period they were subjected to the same amount of PA in the same growth medium (cortical neuronal medium). Therefore, it is reasonable to compare the results obtained with cortical and cerebellar astroglia isolated from the different animals and by the different techniques.

## Conclusion

To our knowledge, this is the first time any of the risk factors for AD, including saturated FFAs, have been shown to provide an experimental explanation for the region-specific damage observed in AD brain. These data warrant further in-depth investigation of the differential FFA metabolic activities in cortical and cerebellar astroglia, which may provide clues that may be translated into potential, novel targets for therapeutic intervention in AD.

## Competing interests

The authors declare that they have no competing interests.

## Authors' contributions

SP conceptualized the study, designed the study protocols and analyzed the collected data. DB and JM participated in the experimental work. DB performed isolation, culture and experimental treatment of primary brain cells. JM carried out western blotting. SP and CC drafted the manuscript. All authors reviewed and approved the final manuscript.
